# A descriptive study of ciguatera fish poisoning in Cook Islands dogs and cats: Treatment and outcome

**DOI:** 10.14202/vetworld.2020.1269-1279

**Published:** 2020-07-06

**Authors:** Michelle J. Gray, M. Carolyn Gates

**Affiliations:** 1 Master of Veterinary Medicine Program, School of Veterinary Science, Massey University, Palmerston North, New Zealand; 2 EpiCentre, School of Veterinary Science, Massey University, Palmerston North, New Zealand

**Keywords:** cats, ciguatera, Cook Islands, dogs, outcome, treatment

## Abstract

**Background and Aim:**

Ciguatera fish poisoning (CFP) is an illness caused by the ingestion of fish containing ciguatoxins. Dogs and cats are susceptible to CFP, but there is little published and much unknown about the condition in these species. This study aimed to document the treatment and outcome of canine and feline cases of CFP, and to look for prognostic indicators.

**Materials and Methods:**

Six years of medical records from the Esther Honey Foundation Animal Clinic (the only veterinary clinic in the Cook Islands during the study period) were reviewed to identify cases of CFP. Data relating to treatment and outcome were collected.

**Results:**

Two hundred and forty-six cases of CFP were identified, comprising 165 dogs and 81 cats. The treatments most commonly administered to cases were fluid therapy and muscle relaxants. Mannitol was only given to five animals. The survival rate was >90% and almost all mortalities occurred in the first week of hospitalization. Recovery was slow, with hospitalization averaging 12.9 days. There was no significant difference in recovery times between dogs and cats. Prolonged periods of anorexia and recumbency were common in both species. Factors associated with prolonged recovery times included case severity, anorexia, and age (in dogs).

**Conclusion:**

This article documented the treatment and outcome of animals afflicted by CFP in the Cook Islands. Therapy for CFP was primarily symptomatic and supportive. The survival rate was high, but recovery was often prolonged. The findings will assist veterinarians in giving prognoses and managing owner expectations.

## Introduction

Ciguatera fish poisoning (CFP) is an illness caused by the ingestion of naturally occurring ciguatoxins. The toxins are produced by microalgae and transferred through the marine food chain. Poisoning occurs after the ingestion of ciguatoxin containing fish [1-3]. Dogs and cats are among the species afflicted by CFP. Indeed, last century, both species were used in bioassays and as test subjects for research into CFP [4-9]. More recently, there have been case reports [10-15], and general articles about the toxicity [16-22]. Objective studies of CFP in dogs and cats are, however, lacking.

The clinical presentation of CFP varies between species [23]. Human CFP is characterized by a combination of gastrointestinal, cardiovascular, and sensory abnormalities [1,2,24]. In contrast, CFP in dogs and cats is characterized by motor impairment, and in particular, by ataxia and paresis [4,7,18]. A detailed description of the symptomology of canine and feline CFP in this case series was presented in an earlier article [25].

There is no specific antidote for ciguatoxin, which acts by binding to voltage sensitive sodium channels [26-28]. Treatment of CFP is, therefore, mostly symptomatic and supportive. Many drugs have been used empirically in human medicine to alleviate gastrointestinal, neurologic, and cardiovascular symptoms [1]. Mannitol is also widely recommended [1] since Palafox *et al*. [29] described the reversal of neurologic symptoms of CFP after mannitol administration. Controlled trials of mannitol therapy for CFP have, however, produced conflicting results and reviewers have argued both for and against its efficacy [30-32].

Treatments for canine and feline CFP have largely been extrapolated from human medicine. Those reported in the literature have included thiamine [14]; steroids, Vitamin B, and “cardiovascular agents” [4]; and atropine, calcium, and lignocaine [9]. However, most of the veterinary case reports attribute patient recovery primarily to nursing care and nutritional support [11,12,16,18]. The treatment protocol of the Esther Honey Foundation (EHF) Animal Clinic (the site of this study) recommends: Decontamination if possible (with emesis and/or activated charcoal), fluid therapy, nutritional support, muscle relaxants, mannitol, nursing care, and symptomatic medication as indicated (e.g., analgesics and gastrointestinal medications) [18].

The prognosis for human CFP is good. Although the toxicity can be debilitating, overall mortality rates are estimated to be <0.1% [33,34]. Fatalities, when they occur, are due to dehydration, cardiovascular shock, or respiratory failure [1,2]. Recovery times can vary from days to months [2,35]. Sensitization can occur after acute CFP in people. This is a phenomenon whereby patients experience a recurrence or aggravation of symptoms following the ingestion of non-toxic fish, food, or drinks. The cause of sensitization is unknown, but hypotheses include bioaccumulation of ciguatoxin and neurologic sensitization [1].

The prognosis for dogs and cats with CFP appears similarly good. While deaths have been reported in animals [13], the majority of (non-experimental) cases have recovered [10-12,14,15]. The duration of illness in dogs and cats is reported to be 1-3 weeks (Table-1). It is unknown whether CFP sensitization occurs in dogs and cats.

**Table-1:** Duration of illness in reports of canine and feline ciguatera fish poisoning.

Reference	Species	Reported time to recovery
Anonymous [10]	Dogs	2 weeks
Cohen [16]	Dogs	2-3 weeks
Forster [18]	Dogs + Cats	Weeks
Bagnis and Fevai [4]	Cats	7-10 days
Hessel *et al.* [6]	Cats	1-5 days
Clark and Whitwell [11]	Cats	2+ weeks
Kemppainen *et al.* [12]	Cats	10 days
Seawright [22]	Cats	2-14 days
Tonge [15]	Cats	2-7 days

This study is the first to specifically evaluate the treatment and outcome of canine and feline CFP cases. A review of current treatment practices is important, both to inform veterinarians faced with treating CFP, and to identify therapies worthy of further investigation. Relying on treatment data from human studies is not appropriate, given the species differences in symptomology. The objective examination of survival rates and recovery times for canine and feline CFP cases is also important, as it will enable veterinarians to give more accurate, evidence-based prognoses for afflicted animals.

This article is the third in a series describing CFP in dogs and cats in the Cook Islands. It aims to describe the treatment and outcome of CFP cases. A second objective is to identify potential prognostic indicators.

## Materials and Methods

### Ethical approval

This retrospective review of case records was deemed to not require ethics approval (Massey University).

### Data collection

The location and methodology of this study is described in detail elsewhere [36]. In brief, the medical records of the EHF Animal Clinic (the only veterinary clinic in the Cook Islands during the study period) were searched for cases with a presumptive diagnosis of CFP. Cases presenting in the 6-year period March 2011-February 2017 were considered for inclusion. Eligible patient files were searched to identify the variables of interest: Treatments administered and details of case outcome (Supplementary Table-1). Data relating to case demographics, exposure history, and clinical signs were also collected [25,36] and used in the survival analyses. Data were collated using Epi-Info software (version 7.2.1.0, CDC, Atlanta, USA).

**Supplementary Table-1:** Variables of interest.

Treatment	Detail
Decontamination	Method
Fluid therapy	Route
Nutritional support	Method
Supplements	Type
Mannitol	
Muscle relaxants	Drug
Sedatives	Drug
Analgesics	Drug
Antibiotics	Drug
Steroids	Drug
NSAIDs	Drug
Eye ointment	Type
Other	Drug
Outcome	
Severity of locomotor dysfunction (mild or severe)^1^	
Severity of respiratory dysfunction (mild or severe)^2^	
Date presented	
Date started eating (if inappetent)	
Date started walking (if recumbent)	
Date discharged/died	
Days anorexic	
Days recumbent	
Days in hospital with CFP	
Outcome (survived, died, euthanized)	
Outcome notes	

1 Mild=No locomotor signs, ataxia or paresis without recumbency; Severe=Sternal or lateral recumbency.

2 Mild=No respiratory signs or tachypnea; Severe=Moderate or marked dyspnea; NSAIDs=Nonsteroidal anti-inflammatory drugs

The age variable was assigned categorical values based on the following criteria:

Juvenile: Age given as ≤12 months; OR animal referred to as a puppy or kitten

Adult: Age given as >12 months and <8 years; OR animal referred to as an adult

Senior: Age given as ≥8 years; OR animal referred to as senior, aged or geriatric

Unspecified: Insufficient detail in the medical record to classify the case as juvenile, adult, or senior

Severity of locomotor dysfunction was classified as mild (no locomotor signs, or ataxia or paresis without recumbency) or severe (sternal or lateral recumbency).

Severity of respiratory dysfunction was classified as mild (no respiratory signs or tachypnea) or severe (moderate or marked dyspnea).

### Statistical analysis

Days anorexic, days recumbent, and days in hospital were calculated by subtracting the presenting date from the date a case started eating (days anorexic); starting walking (days recumbent); and was discharged or died (days in hospital).

Descriptive statistics were performed in Epi-Info. Using the subset of 207 surviving cases with known discharge dates, Kaplan–Meier curves were plotted, and a log-rank test was performed to determine if there was a statistically significant difference in the time to discharge between dogs and cats presumptively diagnosed with CFP. For each species, a survival analysis was then performed using a Cox Proportional Hazards model to identify clinical signs and case characteristics that were associated with the time until discharge. Each variable was screened in a univariate analysis to identify associations with p<0.20 for inclusion in the final multivariable model. A backwards stepwise selection procedure was then used to sequentially remove variables with the highest p-value until all remaining variables in the multivariable model had p<0.05. The significant associations from the multivariable model were reported as hazards ratios with 95% confidence intervals.

## Results

Two hundred and forty-six cases with a presumptive diagnosis of CFP were identified from the 6-year pool of medical records. These comprised of 165 dogs and 81 cats.

### Treatment

Fifteen cases (ten dogs and five cats) received no treatment other than hospitalization, observation, and flea/worm medication. Two hundred and ­thirty-one cases received one or more treatments. The most common therapies administered were fluid therapy and muscle relaxants. Mannitol was the least frequent treatment, with only five cases (four dogs and one cat) receiving an infusion. Table-2 documents the type and frequency of treatments given in this case series.

**Table-2:** Treatments administered to ciguatera fish poisoning cases.

Treatment type	Number of cases	% of cases (n=246)	Specific treatment (number of cases)
Fluids	200	81.3	Intravenous fluids (189); subcutaneous fluids (29); oral fluids (17)
Muscle relaxants	123	50.0	Diazepam (117); methocarbamol (39); midazolam (6)
Other^1^	100	40.6	Maropitant (16), metoclopramide (16), atropine (12), ranitidine (6), cyproheptadine (6), other (54)^2^
Nutritional support	89	36.2	Assisted/syringe feeding (84); nasogastric tube (10); esophageal tube (3)
Analgesics	74	30.1	Buprenorphine (32); morphine (29); butorphanol (19); tramadol (6)
Decontamination	62	25.2	Activated charcoal (60); emesis (4)
Supplements	58	23.6	Potassium (29); B vitamins (22); glucose (10); other (15)^3^
Antibiotics	51	20.7	Amoxiclav (23), amoxicillin (9), cephalexin (8), enrofloxacin (6), metronidazole/spiramycin (6), other (11)
NSAIDs	25	10.2	Meloxicam (14), carprofen (5)
Sedatives	20	8.1	Acepromazine (19); Phenobarbitone (3)
Eye ointment	10	4.1	Lubricant (5), various antibiotic ointments (5)
Steroids	9	3.7	Dexamethasone (7), prednisolone (2)
Mannitol	5	2.0	

1 Excludes flea and worm treatments.

2 No other single medication was given to ≥5 cases. Non-listed treatments included other GIT medications (17), respiratory agents including oxygen (7), skin/ear medications (6), diuretics (4), sedatives (3), and a variety of treatments for secondary or concurrent medical issues (17).

3 Multivitamins were given to seven cases. Other non-listed supplements included nutrigel, amino acid/ electrolyte combinations, coconut oil, honey, and electrolytes

### Outcome

Outcome was specified in 236 cases (95.9%). Five dogs and five cats had no outcome recorded. Of the cases with known outcome, 216 survived (91.5%); 12 died (5.1%); and eight were euthanized (3.4%). Table-3 provides a breakdown of outcome by species.

**Table-3:** Outcome of ciguatera fish poisoning cases.

Outcome	Canine and feline cases (%)	Canine cases (%)	Feline cases (%)
Survived	216 (87.8)	148 (89.7)	68 (84.0)
Died	12 (4.9)	8 (4.8)	4 (4.9)
Euthanized	8 (3.3)	4 (2.4)	4 (4.9)
Unknown	10 (4.1)	5 (3.0)	5 (6.2)
Total	246 (100.0)	165 (100.0)	81 (100.0)

Cause of death was recorded for seven of the 12 cases that died. Five deaths (three dogs and two cats) were due to respiratory arrest (with aspiration suspected in three cases). One cat died during anesthesia for esophageal tube placement; and one dog died from hemorrhagic gastroenteritis (after apparent resolution of CFP).

Reasons given for euthanasia included lack of improvement (one dog and two cats); deterioration (one dog and one cat); and poor clinical state (two dogs). No reason was given for euthanasia of one cat.

### Duration of illness

**Figure-1:**
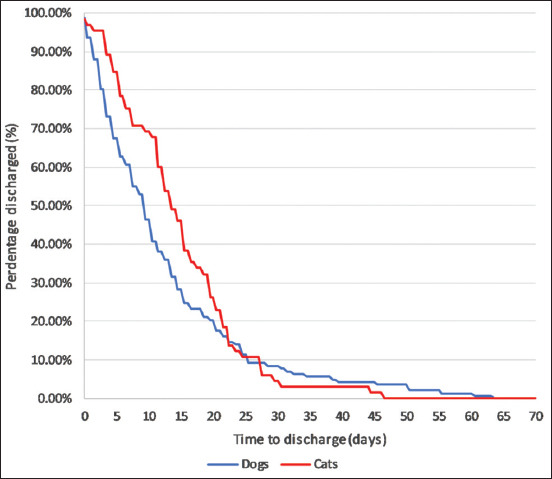
Kaplan–Meier survival curve for time to discharge in dogs and cats with ciguatera fish poisoning.

**Figure-2:**
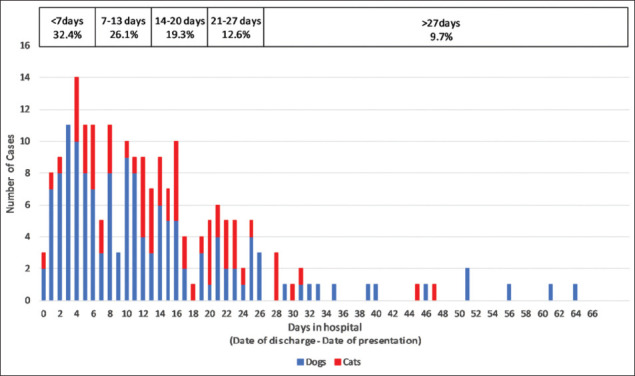
Duration of hospitalization of canine and feline cases of ciguatera fish poisoning: Data from 207 surviving cases.

The duration of hospitalization could be calculated for 207 of the 216 surviving cases (95.8%). Case records of six dogs and three cats failed to specify the date of discharge/recovery. The mean duration of hospitalization was 12.9 days and the median duration 10 days. The longest recorded hospitalization in a dog was 63 days, versus 47 days for a cat. Overall, dogs in this case series recovered slightly quicker (mean 12.3 days, median 9 days, n=142) than cats (mean 14.3 days, median 12 days, n=65). However, when Kaplan–Meier curves were plotted (Figure-1), the difference was not statistically significant (log-rank test, p=0.258). Figure-2 depicts the range of recovery times.

When cases were graded by the degree of locomotor and respiratory dysfunction, severely affected cases took longer on average to recover (Table-4). The difference was most apparent when comparing dogs with mild versus severe locomotor dysfunction.

**Table-4:** Duration of hospitalization: Comparison of cases with mild versus severe locomotor and respiratory dysfunction.

Population	Classification	Number of cases	Days in hospital		

			Mean	Median	Range
Degree of locomotor dysfunction					
All cases	Mild^1^	88	6.6	5	0-23
	Severe^2^	119	17.6	15	0-63
Dogs	Mild^1^	62	5.0	3	0-19
	Severe^2^	80	17.9	15	0-63
Cats	Mild^1^	26	10.4	10	0-23
	Severe^2^	39	16.9	16	1-47
Degree of respiratory dysfunction					
All cases	Mild^3^	156	11.8	10	0-63
	Severe^4^	51	16.2	14	1-51
Dogs	Mild^3^	98	11.1	8	0-63
	Severe^4^	44	15.0	11.5	1-51
Cats	Mild^3^	58	13.1	12	0-45
	Severe^4^	7	24.0	23	8-47

1 Mild=No locomotor signs, ataxia, or paresis without recumbency.

2 Severe=Sternal or lateral recumbency.

3 Mild=No respiratory signs or tachypnea.

4 Severe=Moderate or marked dyspnea

This observation was confirmed by a survival analysis for canine CFP, which identified recumbency, rigidity, and paresis as hazards for prolonged hospitalization. Other clinical signs associated with prolonged illness included dehydration, anorexia, seizures, and hyperesthesia/dysesthesia. The predictive model for time to discharge of canine CFP cases also retained age as a hazard. When compared with the reference group of adult dogs, cases described as senior were 82% less likely to be discharged after any particular duration of hospitalization. Hazard ratios, 95% confidence intervals, and p values are presented in Table-5.

**Table-5:** Cox proportional hazard estimates of the determinants of time to discharge in canine ciguatera fish poisoning cases.

Variable	Hazards Ratio	95% Confidence interval	p-value	Mean duration of hospitalization
Clinical signs^1^				Cases with/without the clinical sign
Recumbency	0.3499	0.226-0.542	0.000003	17 days/5 days
Extensor rigidity	0.4476	0.292-0.687	0.0002	18 days/7 days
Paresis	0.537	0.368-0.783	0.001	15 days/9 days
Dehydration	0.3652	0.186-0.716	0.003	20 days/12 days
Anorexia	0.5643	0.379-0.841	0.005	16 days/8 days
Hyperesthesia/dysesthesia	0.3296	0.130-0.833	0.019	27 days/12 days
Seizures	0.4865	0.237-0.996	0.049	24 days/11 days
Dog age^2^				By age group
(Adult)^3^	1.00	-	-	13 days
Senior^4^	0.1773	0.048-0.649	0.009	29 days
Unspecified^5^	0.5993	0.048-0.649	0.068	13 days
Juvenile^6^	1.0076	0.573-1.772	0.979	9 days

1 Documented at any stage during illness.

2 Reference category in parenthesis.

3 Adult: Age given as >12 months and <8 years; OR animal referred to as an adult.

4 Senior: Age given as ≥8 years; OR animal referred to as senior, aged or geriatric.

5 Unspecified: Insufficient detail in medical record to classify case as juvenile, adult, or senior.

6 Juvenile: Age given as ≤12 months; OR animal referred to as a puppy or kitten

Survival analysis for feline CFP identified tremors, anorexia, and cardiac irregularities as hazards for prolonged illness. Of these, the magnitude of the effect was greatest for cases with cardiac irregularities. Fish exposure was also retained in the predictive model for time to discharge in feline CFP. Compared to the reference group of cats without known fish ingestion, cats that were fed fish by their owners were 3 times more likely to be discharged after any particular duration of hospitalization. Hazard ratios, 95% confidence intervals, and p values are presented in Table-6.

**Table-6:** Cox proportional hazard estimates of the determinants of time to discharge in feline ciguatera fish poisoning cases.

Variable	Hazards Ratio	95% Confidence interval	p-value	Mean duration of hospitalization
Clinical signs^1^				Cases with/without the clinical sign
Tremors	0.4491	0.246-0.819	0.009	18 days/13 days
Anorexia	0.4927	0.287-0.847	0.010	17 days/11 days
Cardiac irregularities	0.31	0.118-0.811	0.017	21 days/14 days
Source of exposure^2^				By source of exposure
(No documented fish exposure)	1.00	-	-	15 days
Fish obtained through neighbor^3^	NA	NA	NA	0 days
Fish obtained through owner	3.6004	1.727-7.506	0.0006	11 days
Fish scavenged^3^	1.3209	0.307-5.691	0.708	12 days
Fish source unspecified^3^	0.5167	0.117-2.281	0.383	28 days

1 Documented at any stage during illness.

2 Reference category in parenthesis.

3 These groups contain ≤3 cases

Recovery times for animals who had repeated episodes of CFP are detailed in Table-7. There was no consistent pattern of either slower or faster recovery during their latter episodes of illness.

**Table-7:** Recovery times of animals with repeated episodes of ciguatera fish poisoning.

Species	Date of CFP 1^st^ episode	Duration of hospitalization (days)	Date of CFP 2^nd^ episode	Duration of hospitalization (days)	Date of CFP 3^rd^ episode	Duration of hospitalization (days)
Cat	August 7, 2012	6	September 8, 2013	23	May 20, 16	Unrecorded
Cat	April 10, 2012	28	June 12, 2014	16		
Cat	January 7, 2013	Unrecorded	April 18, 2013	12		
Dog	December 27, 2011	42	October 24, 2012	1		
Dog	November 29, 2012	15	February 1, 2013	17		
Dog	June 18, 2012	Unrecorded	September 22, 2012	25		
Dog	May 3, 2011	Unrecorded	June 4, 2011	2		
Dog	August 8, 2015	51	March 13, 2016	6		
Dog	August 8, 2015	24	March 14, 2016	11		

CFP=Ciguatera fish poisoning

### Duration of anorexia and recumbency

**Figure-3:**
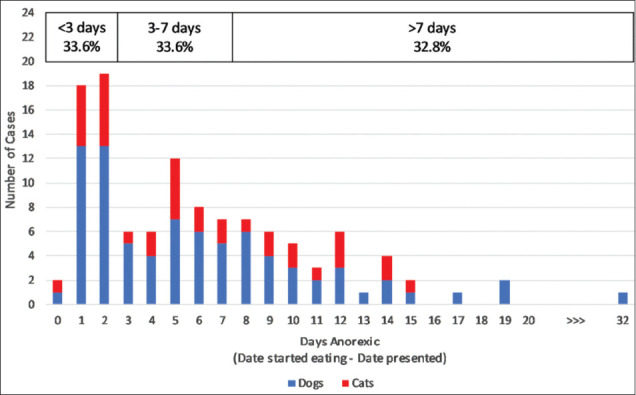
Duration of anorexia in canine and feline cases of ciguatera fish poisoning: Data from 116 cases with anorexia.

One hundred and thirty-three cases had anorexia (55.9% of 238 with recorded clinical signs). It took on average 6.1 days for anorexic animals to start eating voluntarily; the range was 0-32 days. This is based on the data from 116 cases: Data were missing for eight animals that died, and another nine records did not specify the date that eating resumed. Figure-3 depicts the duration of anorexia across all cases. Species differences in the duration of anorexia are presented in Table-8.

**Table-8:** Duration of anorexia and recumbency in ciguatera fish poisoning cases.

Variable	Days anorexic^1^			Days recumbent^2^		
		
Population	Combined	Dogs	Cats	Combined	Dogs	Cats
Number of cases	116	80	36	107	73	34
Mean	6.1	6.1	5.9	11.7	12.5	10.0
Median	5	5	5	10	10	9
Range	0-32	0-32	0-15	0-38	0-38	0-31

1 Days anorexic=Date started eating – date presented.

2 Days recumbent=Date standing/walking – date presented

**Figure-4:**
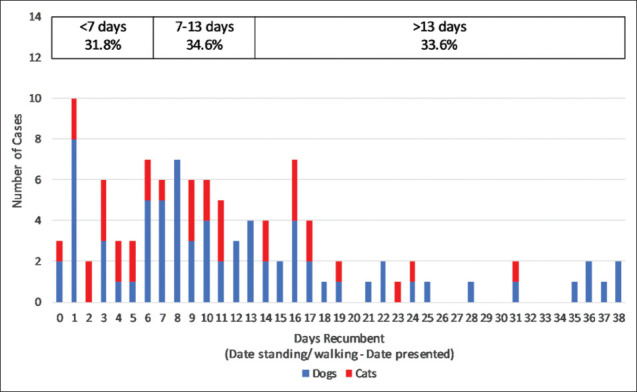
Duration of recumbency in canine and feline cases of ciguatera fish poisoning: Data from 107 cases with recumbency.

One hundred and forty-seven cases were recumbent at some stage of hospitalization (61.8% of 238 with recorded clinical signs). It took on average 11.7 days for recumbent animals to stand and walk, and the range was 0-38 days. This is based on the data from 107 cases: Data were missing for 13 animals that died, and another 27 records did not specify the date the animal start walking. Figure-4 depicts the duration of recumbency across all cases. Species differences in the duration of recumbency are presented in Table-8.

### Time to death or euthanasia

For the 12 cases that died, the mean time to death was 4.5 days (median 3.5 days, range 0-18 days). Only one case (a dog that died from hemorrhagic gastroenteritis) survived longer than a week. All others died in ≤7 days.

For the eight cases that were euthanized, then mean time to euthanasia was 5.0 days (median 4.5 days, range 0-13 days). Only one case (a cat for whom no reason for euthanasia was given), survived longer than a week. All others were euthanized in ≤7 days.

## Discussion

### Study limitations

The results of this study should be interpreted in light of the methodology. Classification of cases was based solely on the attending clinician’s opinion. Therefore, some cases may have been erroneously attributed to CFP, and if so, the recovery and outcome data will be imprecise. Data collection was retrospective, and incomplete cases were included on the grounds that they still contained potentially valuable information. Consequently, the size of some data sets and the statistical power of some analyses are reduced. In addition, the frequency of treatments may be under-reported. A final consideration is that days in hospital were taken to reflect the duration of illness, as it was a measure that could be determined objectively. At the EHF Animal Clinic, cases were generally discharged once eating and walking with only mild ataxia/paresis. It is possible that depending on owner factors, some cases may have been discharged earlier or later in the recovery process.

### Treatment

Decontamination was attempted in only a quarter of the study population. This may be because cases only presented after the onset of clinical signs, i.e., many hours after fish ingestion, and the window of opportunity for decontamination was perceived to have passed. In addition, oral decontamination techniques would have been contraindicated in many cases due to the risk of aspiration (including seven cases that presented with reduced gag, 25 that presented with convulsions or opisthotonos, and 35 that were obtunded [25]). When decontamination was attempted, activated charcoal was the mainstay of therapy. The efficacy of activated charcoal in binding ciguatoxin is, however, unknown. The difficulties of decontamination, combined with the lack of a specific antidote, meant that treatment of CFP cases was primarily symptomatic.

The frequency of therapies used in the study population generally corresponded with the frequency of the clinical signs they targeted (Supplementary Table-2). Fluid therapy and muscle relaxants were most commonly administered (Table-2). These accord with a high frequency of anorexia/dehydration and extensor rigidity/opisthotonos [25]. One discrepancy was the infrequent provision of respiratory support to dyspnoeic patients. This is likely due to a shortage of therapeutic options – oxygen supplies are limited and ventilation not possible at the EHF Animal Clinic. Another exception was nutritional support, which was only provided to 36% of cases despite 56% exhibiting inappetence or anorexia. This discrepancy may be due to animals recovering their appetite before nutritional support was deemed necessary. However, feeding tubes were only utilized in 13 cases despite 38 animals remaining anorexic for more than a week (Figure-3). Possible explanations include a shortage of clinical supplies (feeding tubes/liquid diets); anesthetic concerns (precluding esophageal tube placement); or an oversight of the attending veterinarians.

**Supplementary Table-2:** Frequency of clinical signs observed in ciguatera fish poisoning cases [25].

Clinical signs	Number of reports	Percent^1^
Ataxia	164	68.9
Recumbency	147	61.8
Inappetence/anorexia	133	55.9
Paresis/paralysis/weakness	116	48.7
Hypertonus/extensor rigidity	112	47.1
Tachypnea/dyspnea	109	45.8
Unable to walk	79	33.2
Hindlimbs worse than forelimbs	66	27.7
Opisthotonos	65	27.3
Obtunded mentation	63	26.5
Groaning	62	26.1
Vocalization	60	25.2
Tremors	59	24.8
Nystagmus	51	21.4
Dehydration	46	19.3
Hypersalivation	44	18.5
Proprioceptive deficits	44	18.5
Lethargy	34	14.3
Vomiting	27	11.3
Diarrhea	27	11.3
Cardiac irregularities	23	9.7
Hyperesthesia/dysesthesia	20	8.4
Convulsions/seizures	19	8.0
Abdominal discomfort	16	6.7
No gag reflex	16	6.7
Lacrimation	0	0.0

1 Of n=238 cases in which one or more clinical signs were documented.

Many of the medications administered in this study have not been previously reported (except as part of the EHF Animal Clinic CFP treatment protocol [18]). This is not an indication of new developments in CFP management, but rather an indictment of the existing literature base. Gastrointestinal medications and analgesics are commonly used for the symptomatic treatment of human CFP [1], but this is their first documented use in dogs and cats. Muscle relaxants and sedatives are also reported for the first time in this study. These agents are rarely used in human CFP, but as people experience comparatively little motor dysfunction [1], the difference is not surprising. Of the treatments that have been previously reported to treat canine and feline CFP [4,12], B vitamins, atropine, and steroids were all used in this study, but only in a small number of cases.

The medications administered in this study may have been influenced by factors other than patient assessment. As a charitable foundation, the EHF Animal Clinic relies primarily on donations for stock; therefore, availability may have dictated medication choice. Consequently, no inference can be made from the prevalence of a particular drug within a therapeutic group. The CFP treatment protocol may also have influenced the treatments administered. There is, however, evidence to suggest that clinicians used their individual judgment rather than following the dictates of a protocol. Gastrointestinal medications are mentioned only briefly in the protocol [18], but were frequently administered to cases. In contrast, mannitol is recommended within the protocol, but was only administered to five animals.

The infrequent use of mannitol in the study population is surprising. Mannitol is suggested to reduce both the severity and duration of CFP symptoms in humans and is one of the primary treatments recommended [1]. Extrapolation of the treatment to animals is logical, yet it was administered to only 2% of the study population. For some cases, mannitol therapy would have been contraindicated due to dehydration. In others, the perceived window of opportunity may have elapsed, as mannitol (in humans) must be given within 48-72 h of fish ingestion for optimal efficacy [2,27]. Lack of stock is unlikely to have restricted usage (based on personal experience at the clinic). A final possibility is that veterinarians were unfamiliar with the administration of mannitol, and therefore reluctant to use it. Because the number of cases that received mannitol was so small, no conclusions can be made about its efficacy.

### Outcome

The survival rate in this case series was high (>90%). This tallies with the generally positive outcomes of the previous case reports of CFP, only one of which reported fatalities [13]. The mortality rate in humans is even lower, at <0.1% [34]. This could be due to differences in the availability and standard of medical care for humans versus animals. Alternatively, species differences in pathophysiology may result in a higher mortality rate in dogs and cats. Deaths in this study were most frequently attributed to respiratory failure, a cause of mortality also reported in human CFP [1,2]. However, dogs and cats with CFP manifest more motor dysfunction than humans [25] and therefore, the risk of respiratory muscle fatigue would be greater.

Analysis to detect factors associated with non-survival was not attempted. The small number of non-survivors was deemed insufficient for statistical testing. However, there were two points of note. First, three (37.5%) of the eight animals euthanized, and one (8.3%) of the 12 animals that died were described as senior, compared to an overall proportion of 4.1% seniors. Second, four (20%) of the non-surviving cases suffered flystrike, compared with one (0.5%) of the surviving animals. This suggests that age may be a negative prognostic indicator and that fly-strike may be a portent of impending mortality.

This study did not aim to investigate whether the phenomenon of sensitization occurs in dogs and cats; however, an observation was made during the collection of case data that may be pertinent. One dog was reported to have symptom relapses associated with the ingestion of chicken in the months following her recovery. Chicken has been documented as a food trigger for the recurrence of CFP symptoms in humans [1,27]. While a single, retrospective observation does not prove causation, the possibility warrants investigation given the implications for nutritional management of recovering patients.

### Duration of illness

The duration of illness varied widely across the study population. Some animals were discharged the same day as presentation; however, most (98.5%) were hospitalized. This contrasts with human CFP, where the hospitalization rate is estimated to be 5-12% [37,38]. The discrepancy is most likely due to species differences in pathophysiology. The motor dysfunction seen in canine and feline CFP [25] is more likely to require inpatient management than the sensory dysfunction that predominates in human CFP [1]. Two thirds of cases were hospitalized for more than a week; 40% for more than 2 weeks, and 10% of cases were hospitalized for more than 4 weeks (Figure-2). Because the study population was treated at a charitable clinic, protracted hospitalization of cases was possible even with limited owner finances. The expense of treating cases at a private practice would likely result in “economic euthanasia” of some cases, which would lower the overall survival rate.

The duration of illness did not vary significantly between dogs and cats. This does not necessarily imply that species differences do not exist. They may simply be too small, relative to the overall high degree of variation in recovery times, to be detected in the present study.

It is unsurprising that the duration of illness was longer in more severe cases (Table-4). It is only logical that recovery would take longer when the degree of improvement required is greater. Severity scores were not however included in the predictive models for time to discharge (Tables-5 and 6). This may be because in the survival analysis, a multi-tiered variable that combined both locomotor and respiratory severity was used, rather than a binary mild/severe classification. Further research is needed to determine the relationship between case severity and recovery time.

Factors that were associated with prolonged hospitalization included senior age (dogs); unknown fish exposure (cats); and anorexia (both species). The association with age could be due to a higher frequency of comorbidities in this group; alternatively, older animals may be slower to repair/replace ciguatoxin bound sodium channels. The finding that cats with known fish ingestion recovered faster is perhaps surprising, but could simply reflect more rapid diagnosis and treatment in this population. The association between anorexia and prolonged hospitalization suggests that either anorexia is a consistent marker of severe toxicosis, or that nutrition is important for patient recovery.

A variety of other clinical signs were also included in the predictive models for time to discharge (Tables-5 and 6), presumably because they indicate more severe toxicity. The absence of dyspnea from the models is somewhat surprising as it is thought to correlate with CFP severity [22]. It may be because respiratory dysfunction in CFP is generally short-lived, and therefore does not contribute to the duration of illness. The absence of recumbency in the cat model is also unexpected. It is possible that misclassification of mildly affected cats as recumbent occurred, given the species tendency for torpor when ill. This would make the clinical sign too ubiquitous to be relevant as a risk factor.

### Duration of anorexia and recumbency

Prolonged anorexia and recumbency were common in this case series. The data reported are probably slightly inflated by measurement bias. There is inevitably some delay between changes in a patient’s status and its observation and documentation. Nonetheless, it is notable that of 116 animals with anorexia, two thirds were anorexic for more than 3 days, and a third did not eat for more than a week (Figure-3). Recumbency persisted even longer: Of 107 recumbent cases, two thirds remained that way for more than a week, and a third took more than a fortnight to regain mobility (Figure-4).

Sustained anorexia and protracted recumbency both have implications for case management. Nutritional support was examined in conjunction with other treatments (see above). Data on case management did not, however, extend to the husbandry and nursing care provided. This is a consequence of relying on medical records which seldom included details of nursing activities. Prolonged recumbency, as identified in this study, necessitates good animal husbandry. The recovery of recumbent CFP cases probably depends as much on the standard of nursing as any medication. Aspects of care including bladder management and physiotherapy have the potential to speed recovery, or if neglected, result in complications and prolonged morbidity. The importance of these therapies in determining patient outcome should not be underestimated, despite their omission from the review of treatment.

### Time to death or euthanasia

The first week of hospitalization appears to be a critical period for dogs and cats with CFP. Eighteen of twenty non-survivors died or were euthanized within 7 days of presentation. One of the remaining non-survivors is thought to have died from hemorrhagic diarrhea unrelated to CFP. Thus, for those animals still alive 7 days after the onset of CFP, the prognosis for recovery appears excellent.

## Conclusion

This article documented the treatment and outcome of animals afflicted by CFP in the Cook Islands. Therapy for CFP was primarily symptomatic and supportive. The overall survival rate was high (>90%); and the first 7 days of hospitalization were identified as the critical period for case mortality. Recovery was often prolonged, requiring weeks of hospitalization for the resolution of anorexia and recumbency. Factors associated with prolonged recovery times included case severity, anorexia, and age (in dogs).

The results show that while the prognosis for CFP in dogs and cats is good, patience and persistence are often required for a successful outcome. Veterinarians and owners should be aware of this, and the potential expense incurred by prolonged recoveries, when embarking on treatment of CFP cases. Mannitol therapy has been recommended as a treatment for CFP in people, and a case–control study would be beneficial to determine if it can alleviate the symptoms and/or hasten the recovery of dogs and cats with CFP.

## Authors’ Contributions

MJG designed the study, collected the data, and wrote the manuscript. MCG performed the statistical tests and contributed the associated methods. Both authors read and approved the final manuscript.

## References

[1] Friedman M.A, Fernandez M, Backer L, Dickey R, Bernstein J, Schrank K, Kibler S, Stephan W, Gribble M, Bienfang P, Bowen R, Degrasse S, Flores Quintana H, Loeffler C, Weisman R, Blythe D, Berdalet E, Ayyar R, Clarkson-Townsend D, Swajian K, Benner R, Brewer T.D, Fleming L.E (2017). An updated review of ciguatera fish poisoning:Clinical, epidemiological, environmental, and public health management. Mar. Drugs.

[2] Zlateva S, Marinov P, Yovcheva M, Bonchev G, Ivanov D, Georgiev K (2017). Ciguatera poisoning:Pacific disease, foodborne poisoning from fish in warm seas and oceans. Rev J. IMAB.

[3] Rhodes L.L, Smith K.F, Murray J.S, Nishimura T, Finch S.C (2020). Ciguatera fish poisoning:The risk from an Aotearoa/New Zealand perspective. Toxins.

[4] Bagnis R, Fevai G (1971). La ciguatera feline experimentale a Tahiti. Ciguatera-feline experiments in Tahiti. Rev. Med. Vet.

[5] Bagnis R, Chanteau S, Chengue E, Drollet J.H, Lechat I, Legrand A, Pompon A, Prieur C, Roux J, Tetaria C (1985). Comparison of the Cat Bioassay, the Mouse Bioassay, and the Mosquito Bioassay to Detect Ciguatoxicity in Fish. Proceedings of the Fifth International Coral Reef Congress, Tahiti.

[6] Hessel D.W, Halstead B.W, Peckham N.H (1960). Marine biotoxins I. Ciguatera poison:Some biological and chemical aspects. Ann. N. Y. Acad. Sci.

[7] Kawakubo Y, Kikuchi K (1942). Testing fish poisons on animals and report of a human case of fish poisoning in the South Seas. J. Nav. Med. Sci.

[8] Legrand A, Rentler J.F, Bagnis R (1979). Ciguatera-effets cardiques chez le cat et le rat intoxiques experimentalement. Ciguatera-cardiac effects of experimental intoxication on cat and rat. Rev. Med. Vet.

[9] Legrand A, Lotte C, Bagnis R (1985). Respiratory and Cardiovascular Effects of Ciguatoxin in Cats:Antagonistic Action of Hexamethonium, Atropine, Propanolol, Phentolamine, Yohimbine, Prazosin, Verapamil, Calcium and Lidocaine. Proceedings of the Fifth International Coral Reef Congress, Tahiti.

[10] Anonymous. Small animals-ciguatoxin and ciguatera toxicity (1987). Aust. Vet. Pract.

[11] Clark L, Whitwell G.B (1968). Ciguatera poisoning in cats in Brisbane. Aust. Vet. J.

[12] Kemppainen B, Avgeris S, Jones J.B (2004). Field cases of feline ciguatera. Compend. Contin. Educ. Pract. Vet.

[13] Losacker W (1992). Ciguatera fish poisoning in the Cook Islands. SPC Ciguatera Inf Bull.

[14] Newman A.J (1970). A nervous syndrome in dogs responding to treatment with thiamine. NZ. Vet. J.

[15] Tonge J.I, Battey Y, Forbes J.J (1967). Ciguatera poisoning:A report of two outbreaks and a probable fatal case in Queensland. Med. J. Aust.

[16] Cohen H.Y (2015). Volunteering in paradise. Vet. urs. J.

[17] Dalefield R (2017). Gambierdiscus toxicus. In:Veterinary Toxicology for Australia and New Zealand.

[18] Forster D (2009). Problematic pacific poisonings. Vet. Times.

[19] Fowler M.E (2018). Marine zootoxins. In:Veterinary Zootoxicology.

[20] Lewis R.J Ciguatera (fish poisoning) with special reference to cats. In:Proceedings No. (1987) 103-Veterinary Clinical Toxicology. Post Graduate Committee in Veterinary Science, University of Sydney, Sydney.

[21] McPherson C (1998). Ciguatoxin and tetrodotoxin poisonings in the cat:Their diagnosis, treatment and management. Aust. Vet. Pract.

[22] Seawright A.A (1982). Part 5:Miscellaneous toxicities and toxicants:Ciguatera poisoning. In:Animal Health in Australia.

[23] Banner A.H, Scheuer P.J, Sasaki S, Helfrich P, Alendert C.B (1960). Observations on ciguatera-type toxin in fish. Ann. N. Y. Acad. Sci.

[24] Boucaud-Maitre D, Vernoux J-P, Pelczar S, Daudens-Vaysse E, Aubert L, Boa S, Ferracci S, Garnier R (2018). Incidence and clinical characteristics of ciguatera fish poisoning in Guadeloupe (French West Indies) between 2013 and 2016 :A retrospective cases-series. Sci. Rep.

[25] Gray M.J, Gates M.C (2020). A descriptive study of ciguatera fish poisoning in Cook Islands dogs and cats:Exposure history, clinical signs and formulation of a case definition. Vet. World.

[26] Chinain M, Darius H.T, Gatti C.M, Roué M (2016). Update on ciguatera research in French Polynesia. SPC Fish. Newsl.

[27] Chinain M, Gatti C.M, Roué M, Darius H.T (2019). Ciguatera poisoning in French Polynesia:Insights into the novel trends of an ancient disease. New Microbes New Infect.

[28] Inserra M.C, Israel M.R, Caldwell A, Castro J, Deuis J.R, Harrington A.M, Keramidas A, Garcia-Caraballo S, Maddern J, Erickson A, Grundy L, Rychkov G.Y, Zimmermann K, Lewis R.J, Brierley S.M, Vetter I (2017). Multiple sodium channel isoforms mediate the pathological effects of Pacific ciguatoxin-1. Sci. Rep.

[29] Palafox N.A, Jain L.G, Pinano A.Z, Gulick T.M, Williams R.K, Schatz I.J (1988). Successful treatment of ciguatera fish poisoning with intravenous mannitol. JAMA.

[30] Armstrong P, Murray P, Nesdale A, Peckler B (2016). Ciguatera fish poisoning. N. Z. Med. J.

[31] Friedman M.A, Fleming L.E, Fernandez M, Bienfang P, Schrank K, Dickey R, Bottein M.Y, Backer L, Ayyar R, Weisman R, Watkins S, Granade R, Reich A (2008). Ciguatera fish poisoning:Treatment, prevention and management. Mar. Drugs.

[32] Mullins M.E, Hoffman R.S (2017). Is mannitol the treatment of choice for patients with ciguatera fish poisoning?. Clin Toxicol.

[33] Bravo J, Suárez F.C, Ramírez A.S, Acosta F (2015). Ciguatera, an emerging human poisoning in Europe. J. Aquac. Mar. Biol.

[34] Chan T.Y.K (2016). Characteristic features and contributory factors in fatal ciguatera fish poisoning-implications for prevention and public education. Am. J. Trop. Med. Hyg.

[35] Núñez-Vázquez E, Almazán-Becerril A, López-Cortés D, Heredia-Tapia A, Hernández-Sandoval F, Band-Schmidt C, Bustillos-Guzmán J, Gárate-Lizárraga I, García-Mendoza E, Salinas-Zavala C, Cordero-Tapia A, Núñez-Vázquez E.J, Almazán-Becerril A, López-Cortés D.J, Heredia-Tapia A, Hernández-Sandoval F.E, Band-Schmidt C.J, Bustillos-Guzmán J.J, Gárate-Lizárraga I, García-Mendoza E, Salinas-Zavala C.A, Cordero-Tapia A (2018). Ciguatera in Mexico (1984-2013). Mar. Drugs.

[36] Gray M.J (2020). A descriptive study of ciguatera fish poisoning in Cook Islands dogs and cats:Demographic, temporal and spatial distribution of cases. Vet. World.

[37] Lehane L, Lewis R.J (2000). Ciguatera:Recent advances but the risk remains. Int. J. Food Microbiol.

[38] Pennotti R, Scallan E, Backer L, Thomas J, Angulo F.J (2013). Ciguatera and scombroid fish poisoning in the United States. Foodborne Pathog Dis.

